# Role of the Neutrophil to Lymphocyte Ratio in Guillain Barré Syndrome: A Systematic Review and Meta-Analysis

**DOI:** 10.1155/2022/3390831

**Published:** 2022-09-12

**Authors:** Shirin Sarejloo, Shokoufeh Khanzadeh, Samaneh Hosseini, Morad Kohandel Gargari, Brandon Lucke-Wold, Seyedarad Mosalamiaghili, Pouria Azami, Sanaz Oftadehbalani, Shahram Sadeghvand

**Affiliations:** ^1^Cardiovascular Research Center, Shiraz University of Medical Sciences, Shiraz, Iran; ^2^Student Research Committee, Tabriz University of Medical Sciences, Tabriz, Iran; ^3^Neurosciences Research Center, Tabriz University of Medical Sciences, Tabriz, Iran; ^4^Tabriz University of Medical Sciences, Tabriz, Iran; ^5^University of Florida, Department of Neurosurgery, USA; ^6^Student Research Committee, Shiraz University of Medical Sciences, Shiraz, Iran; ^7^Fatemeh Zahra Hospital, Iran University of Medical Sciences, Tehran, Iran; ^8^Department of Pediatrics, Tabriz University of Medical Sciences, Tabriz, Iran

## Abstract

In this study, we conducted a systematic review and meta-analysis regarding the role of the neutrophil to lymphocyte ratio (NLR) in Guillain Barré syndrome (GBS). The most recent update to the search was on July 18, 2022, through the databases of Web of Science, PubMed, Embase, and Scopus. The Newcastle-Ottawa scale was used for quality assessment of included studies. Finally, 14 studies were included in the review, and among them, ten studies were included in the meta-analysis. Our results showed that NLR levels were significantly increased in the patients with GBS compared with healthy controls (SMD = 1.05; 95%CI = 0.59 to 1.50, *P* < 0.001). After treatment, NLR levels were decreased to the extent that they became similar to healthy controls (SMD = −0.03, 95%CI = −0.29 to 0.22, *P* = 0.204). Moreover, NLR was a stable predictor of outcome or response to treatment in such patients (SMD = 1.01, 95%CI = 0.65 to 1.37, *P* < 0.001); the higher the NLR, the worse the outcome. In addition, patients who underwent mechanical ventilation had higher levels of NLR compared to those who did not (SMD = 0.93, 95%CI = 0.05 to 1.82, *P* = 0.03). However, NLR levels were not different among distinct GBS subtypes, so it could not distinguish among them. In conclusion, our analysis indicates that the NLR levels are highly elevated in patients with GBS. Therefore, the NLR has the potential to be used as a biomarker to inform diagnosis, prognosis, or treatment responses in GBS, and future studies are warranted.

## 1. Introduction

Guillain Barré syndrome (GBS) is a peripheral nervous system immune-mediated disorder marked by muscle weakness [1]. Acute inflammatory demyelinating polyradiculoneuropathy (AIDP) is the most prevalent form of GBS, followed by acute motor axonal neuropathy (AMAN) and acute motor-sensory axonal neuropathy (AMSAN) [1]. The disease has a prevalence of one to two cases per 100,000 people per year, with the majority of patients having an infection prior to the onset of the disorder [2]. Although it is widely assumed that GBS is caused by postinfectious immunological dysfunction that mediates demyelination of the peripheral nervous system, the exact etiology of the disease is yet unknown [2]. Indeed, it is an immunopathologically and clinically complex disorder with a limited number of effective immunomodulatory therapies [3].

Furthermore, there are no biomarkers that can be used to help with disease diagnosis, categorization, or prognosis [4]. These characteristics often contribute to misdiagnosis, overtreatment, treatment failure, and unsatisfactory outcomes. This thereby necessitates identification of disease biomarkers to improve GBS diagnostic and therapeutic outcomes [4]. The neutrophil to lymphocyte ratio (NLR) is a new, inexpensive, simple, widely available, and fast-responding biomarker of cellular immune activation. In addition, it is a valid index of stress and systemic inflammation that has opened a new outlook for clinical medicine. It allows for a better understanding of the biology of inflammation and the coupling between adaptive and innate immunity [5]. During disease, the NLR is disrupted with a shift in balance between adaptive (lymphocytes) and innate (neutrophils) immune responses [5]. Its diagnostic and prognostic usefulness has been investigated in a variety of conditions, including cancer [6], cardiovascular disease [7], and neurological disorders [8, 9]. Several researchers have now looked into the link between NLR and GBS [10–16]. NLR may play a diagnostic and prognostic role in GBS, according to the results of these studies. NLR levels were found to be higher in patients with GBS compared to healthy controls in several studies [10, 12–14]. On the other hand, one recent study found no significant alterations in this marker between GBS patients and controls [11]. In addition, two previous studies declared that NLR could come into use when distinguishing between GBS subtypes [13, 16], while other studies found totally opposite findings [10–12]. This introduces the concept that timing of the test may be important. Furthermore, some previous studies show that early testing may predict the outcome of patients with GBS [11–13]. According to the contradictions in the current data, a systematic review and meta-analysis is required. In this study, we conducted a systematic review of the literature on the role of NLR in GBS and used a meta-analysis to pool the individual data from several studies.

## 2. Method

### 2.1. Search Strategy

**Figure 1:**
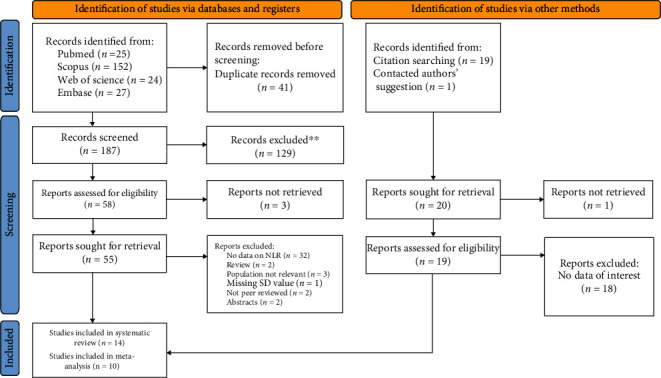
Flowchart of search and study selection.

In compliance with the Preferred Reporting Items for Systematic Review and Meta-Analyses (PRISMA) standards, we performed a comprehensive review and meta-analysis to collect all published papers (Figure 1).

Two reviewers, who were entirely blind to the journal and author details, independently carried out a systematic literature search throughout the online databases of Web of Science, Scopus, PubMed, and Embase. The search strategy was as follows: ((guillain-barre AND syndrome) OR (guillain AND barre AND syndrome) OR GBS) AND ((neutrophil AND lymphocyte AND ratio) OR (neutrophil-to-lymphocyte) OR NLR).

The most recent update to the search was on July 18, 2022. We did not limit our search to a particular language or year of release. To find possibly suitable studies, researchers combed through the reference lists of related reviews and papers. Additionally, the Prospero Registry was combed for information on unpublished and continuing investigations. Because most of the identified papers were conducted in China, we also conducted a rapid nonsystematic search in Google Scholar as a secondary database in English and Chinese to identify grey literature and more relevant studies.

### 2.2. Inclusion and Exclusion Criteria

The following were the criteria for inclusion: (1) studies that are cross-sectional, case-control, or cohort and (2) studies comparing NLR data from GBS patients to healthy controls or studies using NLR data to predict the outcome. Good outcome was defined as Hughes disability score (HDS) < 3 after treatment. The following were the criteria for exclusion: (1) reviews, letters to editors, animal studies, case series, and case reports and (2) studies with similar data.

### 2.3. Extraction of Data and Quality Assessment

Two authors independently investigated the titles/abstracts of the publications obtained. The entire texts of relevant papers were then separately examined for eligibility by the same two authors. A third independent author handled any disagreements between reviewers in both steps.

The first author, year of publication, language, study location, study design, age group (adult or children), total sample size, and the number of cases and controls were collected. NLR level data in GBS cases and controls were all extracted. When there were disagreements, a third author was consulted to reach a consensus.

Two writers independently assessed the quality of the studies included using the Newcastle-Ottawa scale, which has three sections: selection (4 items), comparability (2 items), and outcome (3 items), with a total grade of 0 to 9. Any differences were finally settled by a third author through arbitration.

### 2.4. Statistical Analysis

NLR differences among GBS patients and healthy controls were evaluated using a standardized mean difference (SMD) with a 95% confidence interval (CI). The methods introduced by Wan et al. [17] were used to calculate the mean and SD from the median, range, or IQR. The chi-squared (*χ*^2^) test and the *I*^2^ statistic were used to determine the degree of heterogeneity between study results, and the *I*^2^ statistic was used to quantify inconsistency throughout studies. *I*^2^˃75% and *P* value of *χ*^2^ test ˂ 0.05 were considered significant. The random-effects meta-analysis was chosen in this study because both between-study heterogeneities were significant.

Subgroup analysis was performed according to age group (adults vs. children), study location (Turkey vs. other countries including China and Egypt), and sample size (large studies vs. small studies). We considered studies with sample size of 200 or more as large studies.

For detection of potential publication bias, Funnel plot and Egger's linear-regression test were used, and those with *P* value ˂ 0.05 were considered to have significant publication bias. For statistical analysis, STATA 12.0 software (Stata Corporation, College Station, TX, USA) was used. Statistical significance was defined as a *P* value of less than 0.05.

## 3. Results

### 3.1. Literature Search and Selection

Figure 1 shows the process of identifying and selecting research evidence in this systematic review. In addition to the 228 studies found from the initial database search, 20 further studies were identified through reference lists of relevant articles and Google Scholar and were added. After screening, 14 studies were included in the review [10–16, 18–23]. Among them, ten studies had sufficient data to be included in the meta-analysis [10–16, 22, 24, 25].

### 3.2. Characteristics of the Included Studies

Of the ten studies included in this meta-analysis [10–16, 22, 24, 25], eight studies were retrospective [10–12, 14–16, 24, 25], and two studies were prospective [13, 22]. Concerning document language, all of the documents were in English. Overall, 522 healthy controls and 1207 GBS patients were enrolled in the selected studies. The general characteristics of the selected studies are presented in Table 1. Although the quality assessment of selected studies assessed with the Newcastle-Ottawa scale had different scores ranging from 4 to 9, we included all of them in the meta-analysis (Table 1).

Of the ten studies, five studies compared pretreatment NLR levels in patients with GBS and those of controls [10–14], three studies compared posttreatment NLR levels in patients with GBS and those of controls [11–13], four studies compared pre- and posttreatment NLR levels in patients with GBS [11–13, 15], three studies provided NLR data for both good and poor outcome patients [11–13], and three studies reported association of NLR with mechanical ventilation [22, 24, 25]. Additionally, five studies declared the differences in NLR levels between AIDP and axonal types [10–13, 16], of which three studies showed the differences in NLR levels among AIDP, AMAN, and AMSAN [12, 13, 16].

### 3.3. Meta-Analysis of Differences between GBS Patients and Healthy Controls in NLR Level

**Figure 2:**
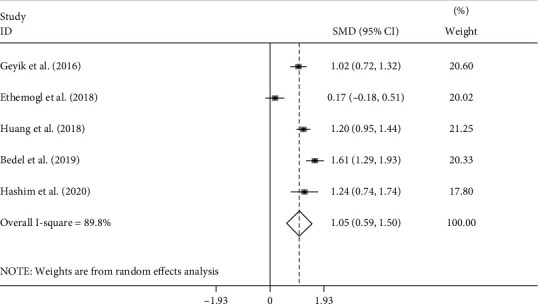
Meta-analysis of differences in NLR levels between GBS patients before treatment and healthy controls.

Before treatment, NLR levels in GBS patients were compared with those of controls in five studies [10–14] with 412 patients with GBS and 522 controls. Compared with the control group, the GBS patients' NLR levels before treatment were significantly higher (random-effects model, SMD = 1.05; 95%CI = 0.59 to 1.50, *P* < 0.001) (Figure 2).

**Figure 3:**
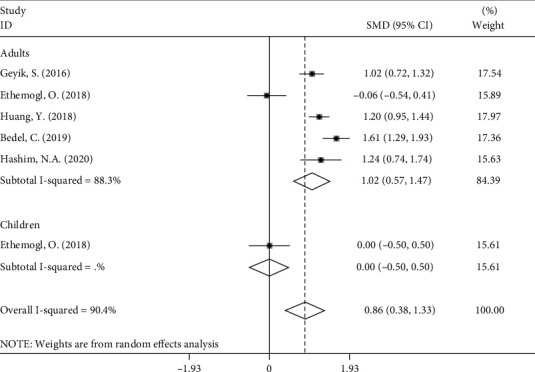
Subgroup analysis of differences in NLR levels between GBS patients before treatment and healthy controls according to age group.

In subgroup analysis according to age group, there were four studies [10, 12–14], including solely the adult participants consisting of 344 patients with GBS and 459 controls. One study [11] included 36 adult and 32 pediatric participants and reported the mean ± SD for both groups. The pooled results showed that the NLR levels in adults with GBS were significantly higher than those in healthy controls (random-effects model, SMD = 1.61, 95%CI = 0.57 to 1.47, *P* < 0.001). The NLR levels of children with GBS in comparison with those of healthy controls showed no significant difference (random-effects model, SMD = 0.00, 95%CI = −0.50 to 0.50) (Figure 3).

**Figure 4:**
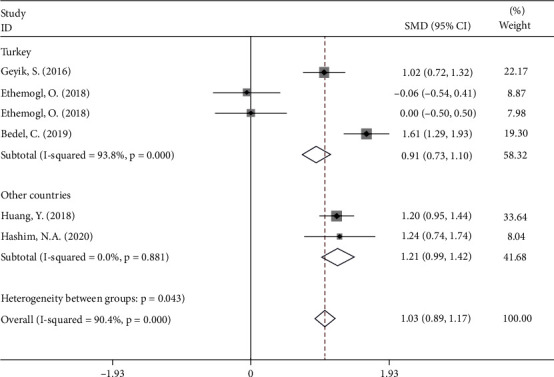
Subgroup analysis of differences in NLR levels between GBS patients before treatment and healthy controls according to study location.

In subgroup analysis according to study location, we found that the NLR levels in patients with GBS were significantly higher than those in healthy controls in both Turkey (SMD = 0.91, 95%CI = 0.73 to 1.10, *P* < 0.001) and other countries (SMD = 1.21, 95%CI = 0.99 to 1.42, *P* < 0.001) (Figure 4).

**Figure 5:**
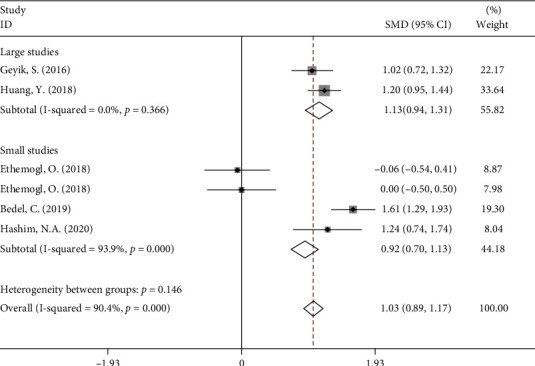
Subgroup analysis of differences in NLR levels between GBS patients before treatment and healthy controls according to sample size.

Subgroup analysis according to age group showed that the NLR levels in patients with GBS were significantly higher than those in healthy controls in either small (SMD = 1.13, 95%CI = 0.94 to 1.31, *P* < 0.001) or large studies (SMD = 1.61, 95%CI = 0.57 to 1.47, *P* < 0.001) (Figure 5).

### 3.4. Association of NLR with Treatment in Patients with GBS

**Figure 6:**
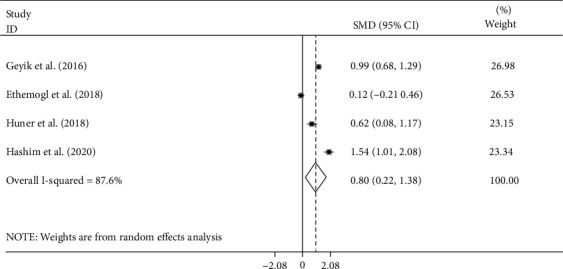
Meta-analysis of differences in NLR levels between pre- and posttreatment GBS.

In the next step, we conducted a comparison of pre- and posttreatment NLR levels of GBS patients based on studies for whom the data (pre- and posttreatment NLR levels) was available. Four studies [11–13, 15], including 224 GBS cases, had sufficient data. The pooled results showed that pretreatment NLR levels were significantly higher than posttreatment NLR levels (random-effects model, SMD = 0.80, 95%CI = 0.22 to 1.38, *P* < 0.001) (Figure 6).

**Figure 7:**
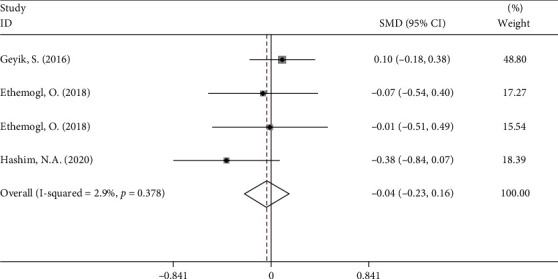
Meta-analysis of differences in NLR levels between GBS patients after treatment and healthy controls.

In addition, NLR levels of GBS patients after treatment became similar to those of controls (fixed-effects model, SMD = −0.04, 95%CI = −0.23 to 0.16, *P* = 0.71) based on three studies [11–13] comprising 197 patients and 204 controls (Figure 7).

### 3.5. Association of NLR with Prognosis in Patients with GBS

**Figure 8:**
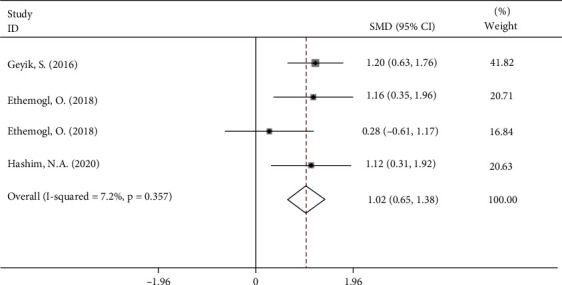
Meta-analysis of differences in NLR levels between GBS patients with good and bad outcome.

Three of the ten studies [11–13] comprising 197 patients evaluated the relationship between NLR and outcome after treatment in patients with GBS. The pooled results showed that patients with bad outcomes had higher levels of NLR compared with good outcome patients after treatment (fixed-effects model, SMD = 1.02, 95%CI = 0.65 to 1.38, *P* < 0.001) (Figure 8). In other words, patients with lower NLR had a better response to treatment in comparison with those with higher NLR.

### 3.6. Association of NLR with Mechanical Ventilation in Patients with GBS

**Figure 9:**
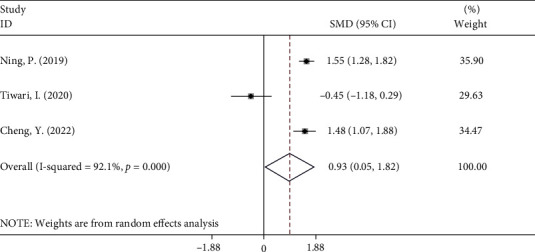
Meta-analysis of differences in NLR levels between GBS patients who underwent mechanical ventilation and those who did not.

Three of the ten studies comprising 706 patients evaluated the relationship between NLR and outcome after treatment in patients with GBS. The pooled results showed that patients who underwent mechanical ventilation had higher levels of NLR compared to those who did not (random-effects model, SMD = 0.93, 95%CI = 0.05 to 1.82, *P* = 0.03) (Figure 9). In other words, patients with lower NLR had a better response to treatment in comparison with those with higher NLR.

### 3.7. Differences in NLR Levels among GBS Subtypes

**Figure 10:**
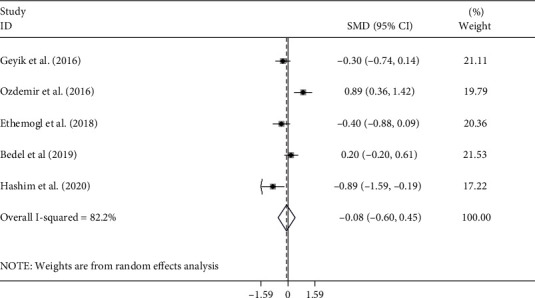
Meta-analysis of differences in NLR levels between patients with AIDP and axonal GBS.

**Figure 11:**
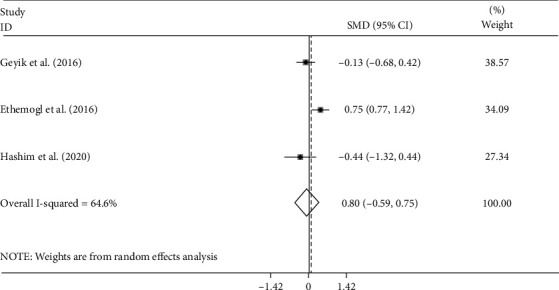
Meta-analysis of differences in NLR levels between patients with AIDP and AMAN GBS.

**Figure 12:**
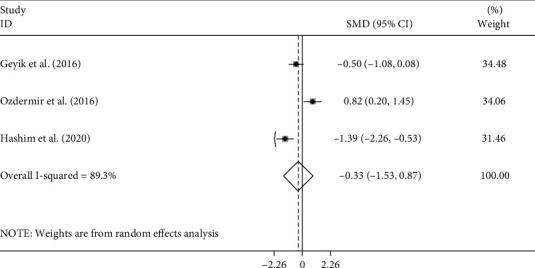
Meta-analysis of differences in NLR levels between patients with AIDP and AMSAN GBS.

**Figure 13:**
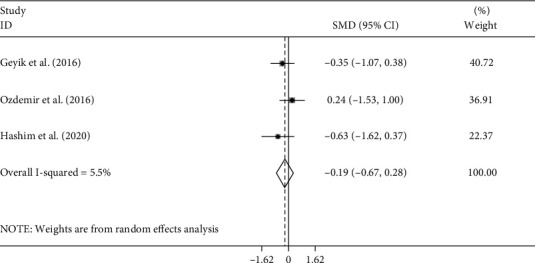
Meta-analysis of differences in NLR levels between patients with AMSAN and AMSAN GBS.

Of the ten studies, five studies [10–13, 16], including 357 patients with GBS, reported the differences in NLR levels between axonal subtypes (including AMAN and AMSAN) and AIDP. Of the five studies, three more detailed studies [12, 13, 16] included 191 patients with GBS, including 117 cases with AIDP subtype and 74 cases with axonal subtype comprising 35 with AMAN and 39 AMSAN and compared these subgroups two-by-two. The pooled results showed that there was no significant difference in NLR levels between AIDP and axonal subtypes (random-effects model, SMD = −0.08, 95%CI = −0.60 to 0.45, *P* = 0.772), AIDP and AMAN (random-effects model, SMD = 0.08, 95%CI = −0.59 to 0.75, *P* = 0.807), AIDP and AMSAN (random-effects model, SMD = −0.33, 95%CI = −1.53 to 0.67, *P* = 0.588), and AMAN and AMSAN (random-effects model, SMD = 0.19, 95%CI = −0.28 to 0.67, *P* = 0.427) (Figures 10–13).

### 3.8. Publication Bias and Small Study Effect

**Figure 14:**
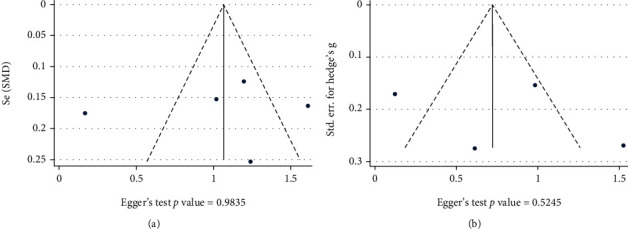
Publication bias between studies on (a) differences in NLR levels between GBS cases and controls and (b) differences between pre- and posttreatment NLR levels in GBS cases.

The results of studies on either difference in NLR levels between GBS cases and controls [10–14] (Figure 14(a)) or differences between pre- and posttreatment NLR levels in GBS cases [11–13, 15] (Figure 14(b)) showed no statistically significant publication bias (Egger's test *P* value = 0.98 and 0.52, respectively).

## 4. Discussion

Our results showed that NLR levels were significantly increased in the patients with GBS compared with healthy controls. After treatment, NLR levels were decreased to the extent that they became similar to healthy controls. Moreover, NLR was a good predictor of outcome or response to treatment in such patients; the higher the NLR, the worse the outcome. In addition, NLR could predict the need for mechanical ventilation. Interestingly, NLR levels were not different among distinct GBS subtypes, so it could not distinguish among them.

In addition to the findings of our meta-analysis, previous studies mentioned some other roles for NLR in GBS. For example, it has been shown that the NLR correlates with disability scores in GBS such as the HDS and the medical research council (MRC) sum scale [12, 13, 18, 20–23]. Sahin et al. revealed that NLR was a predictor of facial diplegia [21] in GBS patients. Also, they mentioned that NLR had a statistically significant correlation with worse nerve conduction studies (NCS) findings such as changes in distal latency, main F latency, conduction velocity, and amplitude [21]. Kim et al. reported that GBS patients who fully recovered without any residual symptoms, compared to those with residual symptoms more than six months after the onset of disease, had significantly lower levels of NLR [19].

NLR is an established marker of inflammation and may reflect an underlying proinflammatory state, as well as immunologic dysfunction, in patients with GBS. Specifically, an elevated NLR may reflect an immune system imbalance [5]. As a brief explanation, neutrophils are a central component of the innate immune system which serves to enhance proinflammatory immune responses to fight pathogens and rid the body of foreign material [26]. On the other hand, lymphocytes are central players in the adaptive immune system, which serves to attenuate proinflammatory responses and regulate immunologic reactions [27]. A relative reduction in adaptive immunity, as reflected by an elevated NLR value, could lead to unregulated proinflammatory responses which contribute in GBS development.

GBS is the leading cause of acute flaccid paralysis. Although the clinical features are varied, patients often present with sensory symptoms and weakness in the legs that advance to the central core muscles and arms [2]. In the absence of adequately specific and sensitive biomarkers, GBS is diagnosed based on the patient's medical history and electrophysiological, neurological, and cerebrospinal fluid investigation [1].

Throughout outbreaks of infectious diseases that cause GBS, the disease prevalence might rise [1]. For example, the COVID-19 virus epidemics have lately been connected to an upsurge in the number of people diagnosed with GBS [28]. Indeed, the outbreak of COVID-19 virus highlighted the lack of universally applicable criteria for the diagnosis and treatment of GBS. It showed the need for more research on this relatively poorly understood disorder [29]. Such investigations are necessary because the diagnosis of GBS is still challenging due to the lack of highly specific and sensitive diagnostic biomarkers, an extensive differential diagnosis, and heterogeneity in clinical presentation [1]. Furthermore, because illness development and outcome differ widely, prognostic indicators for GBS patients must be found [1].

Accordingly, among GBS patients, the role of some biomarkers such as tumor necrosis factor, hypocretin-1, neuron-specific enolase, myelin basic protein, neurofilaments, anti-ganglioside antibodies, neuron-specific enolase, neurofilaments, hypocretin-1, myelin basic protein, chemokines, and complements in disease prognosis and pathology has been established [11]. Furthermore, a recent meta-analysis found that Th1-, Th2-, and Th17-related cytokines were all significantly higher in GBS patients [30]. Although the pathophysiology of GBS is unknown, these findings can be explained by the fact that GBS is caused by an abnormal immune response to infectious pathogens that lead to peripheral nerve injury [30]. Asbury and colleagues explored the role of the inflammatory process in GBS for the very first time in 1969 [31]. They discovered that lymphocyte infiltrates in the nerves in many GBS patients. They also discovered that even in individuals who had healed, persistent inflammation was present, leading them to believe that this is a potential cause of relapse [31]. Since then, a lot of effort has gone into figuring out how the immune system plays a role in inflammation. T cells have been found in the epineurium and endoneurium in sural nerve biopsies of GBS patients, and both CD8+ and CD4+ phenotypes have been detected in these infiltrating T cells [4]. In addition to T cells, there is an elevation in the number of macrophages in the epineurium and endoneurium of these nerves [4].

Furthermore, Yoshii and Shinohara discovered that natural killer cell function was lower in GBS than in the control group [4]; the authors suggested that natural killer cell activity deficiencies could leave people vulnerable to GBS because these cells suppress the immune system [4]. The majority of GBS studies, on the other hand, have concentrated on the diagnostic and prognostic role of cytokines [30]. Not surprisingly, because of the robust immune response, there are changes in cytokine and inflammatory biomarkers' levels in GBS [4].

Considering the involvement of inflammatory processes in hematopoietic multiple-lineage alterations, NLR can be used as an affordable and readily available marker for systemic inflammation [5, 32]. It is, in fact, the number of neutrophils divided by the total number of lymphocytes and is widely employed as a reliable and easily accessible biomarker for multiple conditions [5, 32]. The average range of NLR in adults is 1-2, with values greater than 3.0 and less than 0.7 being abnormal [5]. NLR in the 2.3-3.0 range may act as an early warning sign for pathological conditions or processes such as cancer, atherosclerosis, mental disorders, and neurologic illnesses [5]. It has also been proven in multiple studies to be a highly sensitive sign for infection [33], inflammation, and sepsis [34]. In critical illness or acute disease, NLR should be evaluated on a daily basis, with absolute values and dynamic course being monitored [5]. It should be utilized on a regular basis in emergency rooms, intensive care units, and acute medicine settings such as surgery, orthopedics, traumatology, cardiology, neurology, psychiatry, and even on cancer wards [5].

Zahorec, in 2021, postulated that elevated NLR values are secondary to a multifactorial process involving neuroendocrine and immunologic input [5]. Stress and severe illness can activate the hypothalamic-pituitary-adrenal (HPA) axis leading to elevations in cortisol that stimulate neutrophil demargination and maturation, as well as lymphocyte apoptosis [35–39]. Immunologically, severe illness increases the production of neutrophils from the bone marrow and can lead to lymphopenia via various proposed mechanisms [40–42]. Ultimately, a relative neutrophilia and lymphopenia can result, leading to an elevated NLR.

The findings in this report are subject to at least four restrictions. First, there was significant statistical heterogeneity across studies, as previously stated. This is most likely due to variations in study inclusion criteria, recruiting conditions, and target population. Furthermore, the majority of the studies included did not give information on the stage of GBS. As a result, the varied phases of patients across studies may have contributed to the between-study heterogeneity reported in this meta-analysis (Table 2).

Nonetheless, these findings underscore the necessity for ongoing research into the NLR level in GBS patients, as well as the control of pertinent clinical and methodological variables. This should be done in order to better understand the disease's cause. Another limitation was that the data retrieved from the relevant papers did not allow for the testing of the relationship between NLR and facial diplegia (a common symptom of GBS) and patients' mortality. As a result, these are critical topics that should be investigated further.

In conclusion, this is the first systematic review and meta-analysis to assess the NLR levels in patients with GBS. Our analysis indicates that the NLR levels are highly elevated in patients with GBS. Therefore, the NLR has the potential to be used as biomarkers to inform diagnosis, prognosis, or treatment responses in GBS, and future studies are necessary to validate this hypothesis.

## Figures and Tables

**Table 1:** General characteristics of studies included in the meta-analysis.

First author	Year	Design	Region	Age group	Comparison between cases and controls					Comparison between GBS types								Comparison between GBS cases with good and bad outcome				Comparison between GBS cases with and without mechanical ventilation				NOS score
					Healthy control		GBS cases																			
										AIDP type		Axonal type						Good outcome		Bad outcome		MV		Non-MV		
												AMAN		AMSAN		Total										
					*N*	NLR	*N*	Pretreatment NLR	Posttreatment NLR	*N*	NLR	*N*	NLR	*N*	NLR	*N*	NLR	*N*	NLR	*N*	NLR	*N*	NLR	*N*	NLR	
Geyik	2016	R	Turkey	Adult	101	2.53 ± 0.94	94	5.43 ± 3.98	2.61 ± 0.69	64	5.41 ± 3.09	16	5.82 ± 3.09	14	7.04 ± 3.98	30	6.38 ± 3.50	78	4.67 ± 3.57	16	9.01 ± 3.89	—	—	—	—	6
Ozdemir	2016	R	Turkey	Adult	—	—	62	—	—	35	5.78 ± 5.23	12	3.36 ± 1.20	15	2.15 ± 0.54	27	2.24 ± 0.83		—	—	—	—	—	—	—	8
Ethemoglu	2018	R	Turkey	Adult and children	63	2.70 ± 1.17	68	2.93 ± 1.53	2.75 ± 1.35	40	2.53 ± 1.44	—	—	—	—	28	3.08 ± 1.32	15	1.97 ± 0.87	53	2.98 ± 1.45	—	—	—	—	5
Huang	2018	R	China	Adult	217	1.51 ± 0.56	117	2.90 ± 1.81	—	—	—	—	_	—	—	—	—	—	—	—	—	—	—	—	—	6
Huner	2018	R	Turkey	Children	—	—	27	3.29 ± 4.30	1.37 ± 0.71	—	—	—	—	—	—	—	—	—	—	—	—	—	—	—	—	8
Bedel	2019	R	Turkey	Adult	101	1.29 ± 0.52	98	3.77 ± 2.13	—	59	3.94 ± 2.27	—	—	—	—	39	3.51 ± 1.89	—	—	—	—	—	—	—	—	6
Ning	2019	R	China	Adult	—	—	—	—	—	—	—	—	—	—	—	—	—	—	—	—	—	74	7.33 ± 5.42	352	2.92 ± 1.91	6
Hashim	2020	P	Egypt	Adult	40	2.58 ± 0.85	35	3.95 ± 1.34	2.27 ± 0.76	18	3.47 ± 1.04	7	4.04 ± 1.85	10	4.94 ± 1.08	17	4.56 ± 1.39	26	3.60 ± 1.18	9	5.11 ± 1.78	—	—	—	—	6
Tiwari	2020	P	India	Children	—	—	—	—	—	—	—	—	—	—	—	—	—	—	—	—	—	9	1.37 ± 0.89	37	1.91 ± 1.27	7
Cheng	2022	R	China	Adult	—	—	—	—	—	—	—	—	—	—	—	—	—	—	—	—	—	30	7.72 ± 8.58	204	2.77 ± 1.53	7

AIDP: acute inflammatory demyelinating polyradiculoneuropathy; AMAN: acute motor axonal neuropathy; AMSAN: acute motor-sensory axonal neuropathy; R: retrospective; P: prospective; NLR: neutrophil to lymphocyte ratio; MV: mechanical ventilation.

**Table 2:** General characteristics of studies included merely in qualitative review.

First author	Year	Design	Region	Age group	Main findings	NOS score
Gumusyayla	2017	R	Turkey	Adults	NLR were found to be significantly higher in GBS patients participating in the study than healthy volunteers. In addition, NLR was positively correlated with the Hughes score and negatively correlated with the MRC sum score calculated at the time of admission and three months after admission.	6
Sahin	2017	R	Turkey	Adults	NLR was a predictor of facial diplegia in GBS patients. Also, NLR had a statistically significant correlation with MRC and NCS findings such as distal latency, main F latency, conduction velocity, and amplitude.	6
Kim	2019	R	Korea	Children	NLR was not different between GBS patients with full recovery and those with functional deficit after treatment.	6
Tunc	2019	R	Turkey	Adults	NLR was significantly correlated with HDS at the end of the first month after GBS.	7

R: retrospective; P: prospective; NLR: neutrophil to lymphocyte ratio; NCS: nerve conduction studies; HDS: Hughes disability score; A: adults; C: children.

## Data Availability

The dataset supporting the conclusions of this article is included within the article.

## Abbreviations

GBS: Guillain Barré syndrome
AIDP: Acute inflammatory demyelinating polyradiculoneuropathy
AMSAN: Acute motor-sensory axonal neuropathy
NLR: Neutrophil to lymphocyte ratio
PRISMA: Preferred Reporting Items for Systematic Review and Meta-Analyses
HDS: Hughes disability score
NOS: Newcastle-Ottawa scale
SMD: Standardized mean difference
95% CI: 95% confidence interval
MRC: Medical research council
NCS: Nerve conduction studies.
